# Supplementary material for: 13-Decyl Berberine Derivative Is a Novel Mitochondria-Targeted Antioxidant and a Potent Inhibitor of Ferroptosis
Source: Cells. 2025 Dec 10;14(24):1963. doi: 10.3390/cells14241963 (PMC12732116; doi:10.3390/cells14241963)
Supplement: Supplementary file 1 [file cells-14-01963-s001.zip › cells-3984968-supplementary.pdf]

Supplement figures:

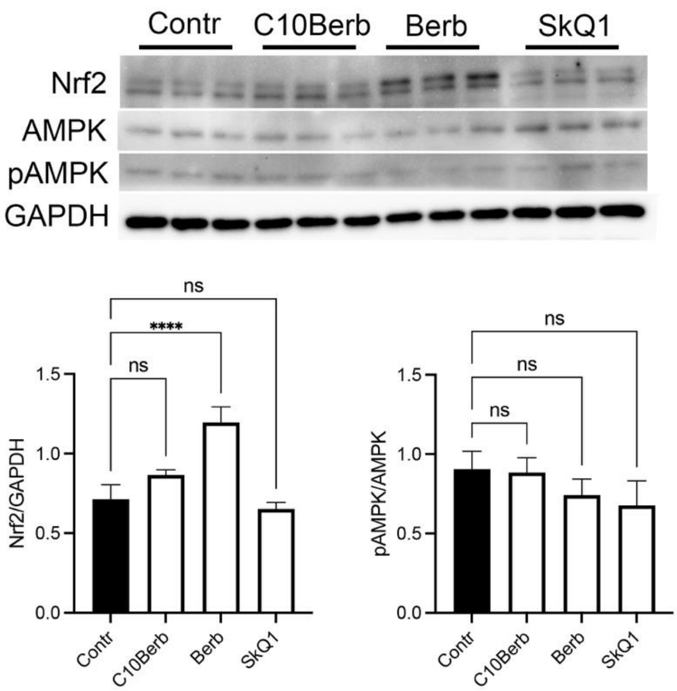

Fig.S1. Effect of C10Berb (100 nM), berberine (Berb, 10 mkM), and SkQ1 (100 nM) on Nrf2, AMPK, phosphoThr172--AMPK (pAMPK), and GAPDH protein levels. Primary human fibroblasts were incubated with the specified concentrations of compounds for 24 hours. The results were analyzed using Image Lab v. 6.0.0. N=3, $***p \leq 0.001$

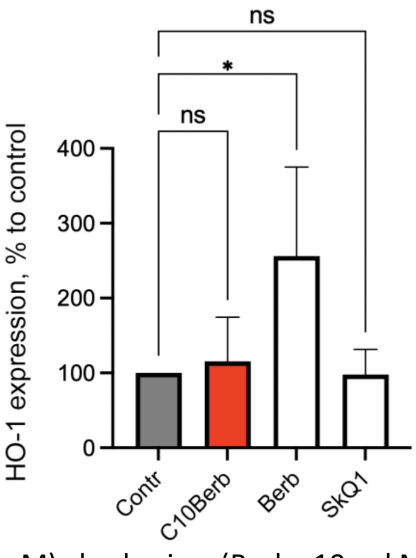

Fig.S2. Effect of C10Berb (100 nM), berberine (Berb, 10 mkM), and SkQ1 (100 nM) on the expression level of heme oxygenase-1 (HO-1). Primary human fibroblasts were incubated with the specified concentrations of compounds for 24 hours. Results of RT PCR. N=3, $*p < 0.05$

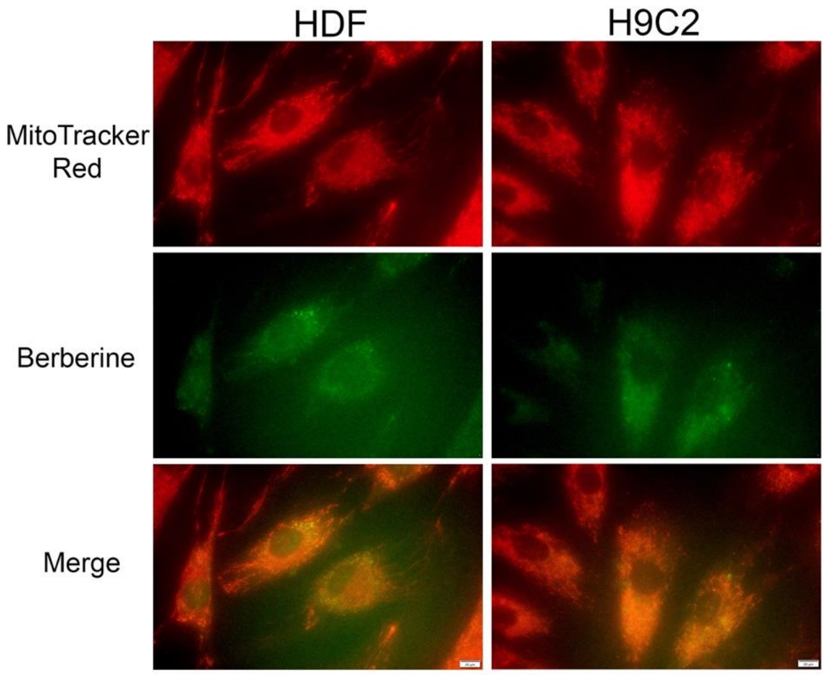

Fig.S3. Accumulation of C10Berb and berberine in H9c2 cardiomyocytes and in human diploid fibroblasts (HDF). Localization of Berberine in H9C2 cells or in HDF was analyzed after incubation with 10 µM Berberine for 1 h followed by staining of mitochondria with MitoTracker Red (100 nM, 15 min). Fluorescence was analyzed using a fluorescence microscope Olympus IX 83 (Tokyo, Japan). Bar – 20 µm.

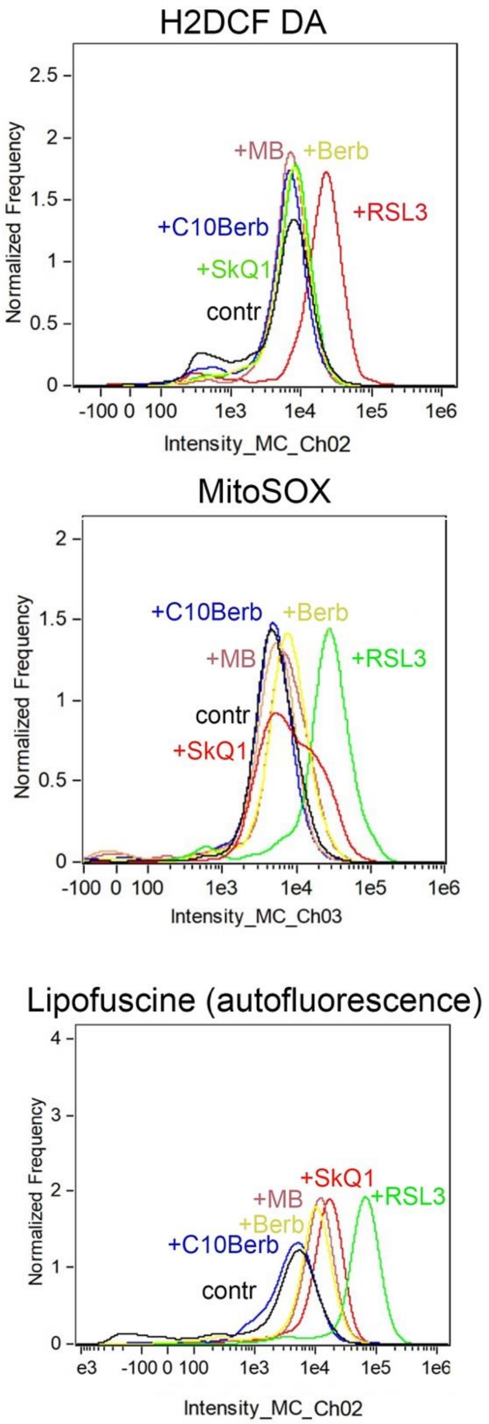

Fig.S4. Representative flow cytometry plots for H9C2 cells corresponding to Figure 5. These plots demonstrate a normal distribution of fluorescence across the cell population in all cases.
